# Fasting plasma glucose is an independent predictor for severity of H1N1 pneumonia

**DOI:** 10.1186/1471-2334-11-104

**Published:** 2011-04-21

**Authors:** Wei Wang, Hong Chen, Qiang Li, Bo Qiu, Jiajun Wang, Xiwen Sun, Ying Xiang, Jinchao Zhang

**Affiliations:** 1 Department of Endocrinology, Second Hospital of Harbin Medical University, Harbin 150086, China; 2 Department of Respirology, Second Hospital of Harbin Medical University, Harbin 150086, China; 3 Department of Neurosurgery, First Hospital of China Medical University, Shenyang, 110001, China; 4 Department of Computer, Second Hospital of Harbin Medical University, Harbin 150086, China; 5 Department of Cancer epidemiology research, Cancer Research Institute of Harbin Medical University, Harbin 150086, China

**Keywords:** Influenza A (H1N1), Pneumonia, Fasting plasma glucose (FPG)

## Abstract

**Background:**

The pandemic influenza A (H1N1) virus emerged during 2009 and has spread worldwide. This virus can cause injuries to the lungs, liver, and heart. However, data regarding whether this influenza virus can affect pancreatic islets are limited. We investigated the effects of influenza A (H1N1) pneumonia on fasting plasma glucose (FPG) and islet function, and evaluated possible correlations between biochemical test results and the severity of H1N1 pneumonia.

**Methods:**

We performed a retrospective analysis of patients either diagnosed with or suspected of having H1N1 pneumonia who were admitted to our hospital in 2009. Possible associations between FPG levels and H1N1 virus infection were assessed by logistic regression. Correlation and regression analyses were used to assess relationships between FPG and biochemical test results. Associations between admission days and significant data were assessed by single factor linear regression. To evaluate effects of H1N1 on pancreatic β-cell function, results of a resistance index (homa-IR), insulin function index (homa-β), and insulin sensitivity index (IAI) were compared between a H1N1 group and a non-H1N1 group by t-tests.

**Results:**

FPG was significantly positively associated with H1N1 virus infection (OR = 1.377, 95%CI: 1.062-1.786; p = 0.016). FPG was significantly correlated with AST (r = 0.215; p = 0.039), LDH (r = 0.400; p = 0.000), BUN (r = 0.28; p = 0.005), and arterial Oxygen Saturation (SaO_2_; r = -0.416; p = 0.000) in the H1N1 group. H1N1 patients who were hypoxemic (SaO_2_<93%) had higher FPG levels than those who were not hypoxic (9.82 ± 4.14 vs. 6.64 ± 1.78; p < 0.05). FPG was negatively correlated with SaO_2 _in the H1N1 group with hypoxia (SaO_2_<93; r = -0.497; p = 0.041). SaO_2 _levels in patients with high FPG levels (≥7 mmol/L) were significantly lower than those of H1N1 patients with low FPG levels (<5.6 mmol/L). There were no significant differences in homa-IR, homa-β, or IAI between the H1N1 and non-H1N1 groups after adjusting for age, sex, and BMI.

**Conclusions:**

FPG on admission could be an independent predictor for the severity of H1N1 pneumonia. Elevated FPG induced by H1N1 pneumonia is not a result of direct damage to pancreatic β-cells, but arises from various factors' combinations caused by H1N1 virus infection.

## Background

The pandemic influenza A (H1N1) virus emerged in the United States and Mexico last year and rapidly spread worldwide [1,2]. Up to December 13, 2009, more than 208 countries and overseas territories or communities have reported laboratory-confirmed cases of pandemic influenza H1N1 2009, which has caused at least 10,582 deaths [3]. It has been indicated that this virus can induce pulmonary complications, respiratory failure, and liver and heart injuries. Diabetes, which is a major risk factor for H1N1 infection, has been frequently observed among severe cases and those who died [4-6]. However, data regarding whether the influenza virus can affect the plasma glucose level and islet function remain limited.

Based on the data from H1N1 cases in our hospital, we decided to explore possible relationships between fasting plasma glucose (FPG) levels, relevant laboratory test findings, and the clinical course of H1N1 infection. In addition, considering the possible effects of viral damage on pancreatic β-cell function, we compared changes of islet function between confirmed H1N1 patients and non-H1N1 patients.

## Methods

### Patients

Data were collected from suspected H1N1-infected patients who had complications of pneumonia in the Emergency Ward of the Second Hospital of Harbin Medical University in 2009. The diagnostic criteria for suspected H1N1, which were similar to those of the Centers for Disease Control and Prevention (CDC) criteria [7], were defined by the Chinese Ministry of Health: fever, known exposure to H1N1, respiratory symptoms, and infiltrates observed on chest radiography [8]. Pharynx swab samples of all suspected cases were sent to the provincial CDC, where H1N1 virus infection was detected and confirmed by real time PCR.

In this retrospective analysis, the clinical and biochemical characteristics of 34 patients positive for H1N1 and 67 patients with non-H1N1 pneumonia were compared. Patients were excluded if they were younger than 16 years of age, were pregnant, had a history of diabetes, or died. Data from laboratory examinations were collected on admission and before steroid treatment, including FPG, glycosylated hemoglobin (HBA1c), function tests for liver and kidney, myocardial enzymes, blood cells, and arterial blood gas analysis. Arterial oxygen saturation (SaO_2_) < 93% (measured on supplemental oxygen) was also used to classify cases as 'serious'; the duration of a hospital stay was the main indicator to judge severity of the disease. Plasma insulin (FINS) was randomly tested among 21 H1N1-positive and 31 H1N1-negative patients at the same time of FPG determinations.

To account for possible H1N1 effects on pancreatic β-cell function, a homeostasis model assessment (Homa) and IAI were used to measure insulin resistance and sensitivity [9,10]. These included: an insulin resistance index: Homa-IR = Ln(FPG × FINS/22.5); β-cell function index: Homa-β = 20 × FINS/(FPG-3.5); and insulin sensitivity index : IAI = -Ln(1/(FPG × FINS). This study was approved by the Ethics Committee of the Harbin Municipal Bureau of Health.

### Treatments

Upon admission, all patient blood samples had been screened with hematology, biochemistry, and virology tests. All patients were empirically administrated antivirals, broad-spectrum antibiotics, and mechanical ventilation therapy before certification of viral or bacterial infections. Baseline and serial chest radiographs and CT scans, if necessary, were performed for all suspected or confirmed H1N1 patients. For suspected or confirmed H1N1 patients with severe pneumonia, as indicated by persistent fever and deteriorating lung opacities on chest X-ray, intravenous therapy with methylprednisolone (120-500 mg every day for 5 days followed by a decreasing dose based on the patient's condition) was used as necessary treatment.

### Statistical analysis

Results are given as means ± SDs or percentages and compared between different groups by independent t-tests or ANOVA, as appropriate. Associations between FPG and H1N1 virus infection, expressed as Odds Ratios (OR) and 95% confidence intervals (CI), were examined by logistic regression analysis after controlling for sex, age, and BMI. Correlation analysis was used to evaluate possible relationships between FPG and biochemical test results. Associations between number of admission days and significant data among the two groups were further assessed by single factor linear regression. Associations between FPG and SaO_2 _were assessed by correlation and regression analyses. To evaluate possible H1N1 effects on pancreatic β-cell function, homa-IR, homa-β, and IAI were compared by *t*-tests between H1N1 and non-H1N1 groups after adjusting for age, gender, and BMI. All analyses used SPSS version 13.0 (SPSS Inc., Chicago, IL, USA). P < 0.05 (two-tailed) was considered to be statistically significant.

## Results

### 1. Comparisons of clinical features between H1N1 and non-H1N1 pneumonia patients

All data for the patients diagnosed as suspected H1N1 and confirmed H1N1 were collected in the Emergency Ward of the Second Hospital of Harbin Medical University in 2009. All patients were Han Chinese, except for one Man Chinese, and all were from Harbin City or surrounding areas. Table 1 summarizes the clinical and biochemical characteristics of the H1N1 and non-H1N1 patients. There were no statistically significant differences for age, gender, or BMI between the two groups. There were no detectable differences in WBC counts and differential counts, and hepatic and renal functions between the two groups.

**Table 1:** Clinical and laboratory Data of study subjects

	Patient with non-H1N1 pneumonia (n = 67)	Patients with confirmed H1N1 pneumonia (n = 34)	Odds Ratio (95%CI) for H1N1	P Value
Age (years)	33.70 ± 13.32	37.76 ± 12.59		
Male (%)	53.7	55.9		
BMI	24.98 ± 4.75	24.54 ± 3.69		
Duration of hospitalization (days)	11.91 ± 8.77	17.65 ± 8.28**		
Mechanical ventilation(no.)	12	18		
Excluded patient(no.)	4	7		
Pregnant women	1	3		
Diabetes of history	3	2		
Death	0	2		
White cell count (×10^9^/L)	7.89 ± 5.90	8.84 ± 5.95	1.011(0.984-1.040)	0.417
Neutrophils (×10^9^/L)	4.67 ± 2.78	5.86 ± 3.68	1.121(0.938-1.339)	0.208
Lymphocytes (×10^9^/L)	1.16 ± 0.56	0.87 ± 0.63	0.367(0.120-1.121)	0.078
Monocytes (×10^9^/L)	0.51 ± 0.32	0.42 ± 0.36	0.358(0.058-2.205)	0.268
ALT (U/L)	81.53 ± 139.48	53.39 ± 43.59	0.997(0.990-1.003)	0.309
AST (U/L)	102.54 ± 183.13	122.82 ± 145.68	1.000(0.998-1.003)	0.704
CK (U/L)	190.68 ± 225.53	345.86 ± 635.14	1.001(1.000-1.002)	0.079
CK-MB (U/L)	22.32 ± 18.81	19.55 ± 13.30	0.995(0.953-1.038)	0.811
LDH (U/L)	501.59 ± 613.70	1139.63 ± 1086.24**	1.001(1.000-1.002)	0.010*
BUN (μmol/L)	4.54 ± 2.42	6.57 ± 4.46*	1.184(1.024-1.369)	0.022*
CREA (μmol/L)	76.93 ± 35.19	92.97 ± 52.57	1.009(0.998-1.019)	0.119
FPG (mmol/L)	6.13 ± 1.54	7.93 ± 3.44**	1.377(1.062-1.786)	0.016*
PO_2 _(mmHg)	79.42 ± 31.73	70.29 ± 36.53	1.003(0.985-1.021)	0.729
PCO_2 _(mmHg)	37.10 ± 4.79	35.86 ± 5.20	0.948(0.856-1.046)	0.280
SaO_2 _(%) ^**&**^	93.23 ± 7.30	86.29 ± 13.23*	1.045(0.976-1.118)	0.205

*p < 0.05; **p < 0.01, vs. non-H1N1 patients; *significant exam associated with H1N1 infection assessed by logistic regression after controlling age, sex and BMI. The data expressed as mean ± SD, or percentage, or OR and 95%CI

However, significant differences of biochemical exam results were found in plasma glucose levels (7.93 ± 3.44 vs. 6.13 ± 1.54, H1N1 vs. non-H1N1; p < 0.01), LDH (1139.63 ± 1086.24 vs. 501.59 ± 613.70, H1N1 vs. non-H1N1; p < 0.01), BUN (6.57 ± 4.46 vs. 4.54 ± 2.42, H1N1 vs. non-H1N1; p < 0.05), and SaO_2 _(86.29 ± 13.23 vs. 93.23 ± 7.30, H1N1 vs. non-H1N1; P < 0.05). The number of admission days for H1N1 patients were obviously longer than for non-H1N1 patients (17.65 ± 8.28 vs. 11.91 ± 8.77, H1N1 vs. non-H1N1; p < 0.01). Logistic regression analysis indicated significant positive associations between FPG (OR = 1.377, 95%CI:1.062-1.786; p = 0.016), LDH (OR = 1.001, 95%CI: 1.000-1.002; p = 0.010), BUN (OR = 1.184, 95%CI: 1.024-1.369; p = 0.022) and H1N1 virus infection.

### 2. Relationship between FPG and H1N1

**Figure 1:**
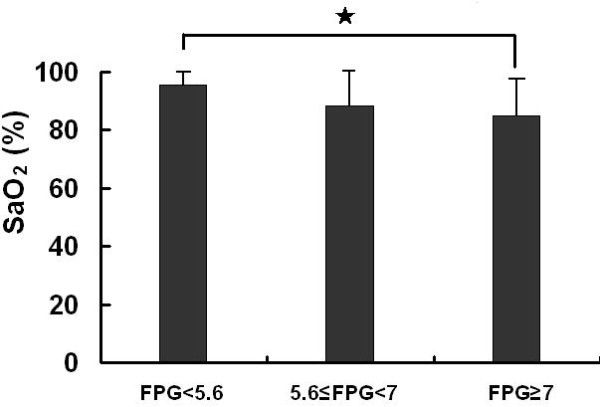
The comparison of SaO_2 _among different levels of FPG in H1N1 group *, p < 0.05.

**Figure 2:**
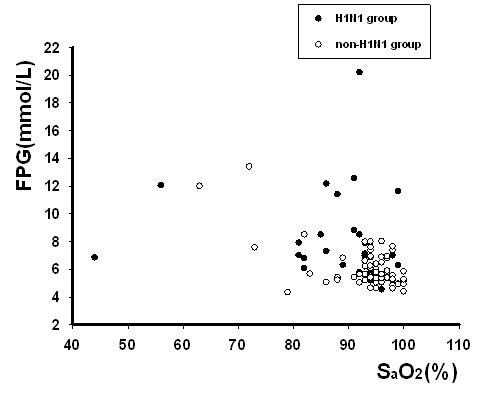
**The relationship between FPG and SaO_2 _among H1N1 and non-H1N1 group evaluated by scatter graph**.

**Figure 3:**
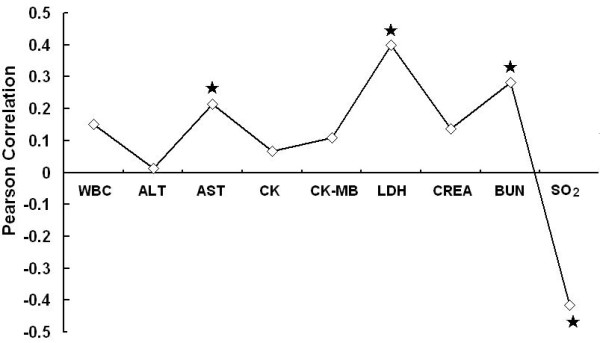
**Correlation analysis between FPG and biochemical exams among H1N1 patients**. *significant correlation with FPG.

Defining SaO_2 _of 93% as a threshold, we further investigated the relationship between FPG and SaO_2_. Those with high FPG levels (≥7 mmol/L) had a significantly lower SaO_2 _level than those with low FPG levels (<5.6 mmol/L) among H1N1 patients (Figure 1). The association between FPG and SaO_2 _is shown in Figure 2. In the H1N1 group, patients with hypoxia (SaO_2_< 93%) had remarkably higher FPG levels than those of patients with SaO_2 _≥ 93% (Table 2). Elevated FPG also paralleled a trend for prolonged hospitalization, although there was no correlation between FPG and hospitalization duration (data not shown). Figure 3 shows the relationships between FPG levels and other biochemical indicators in the H1N1 group. Significant Pearson correlation coefficients were found between FPG and AST (r = 0.215; p = 0.039), LDH (r = 0.400; p = 0.000), BUN (r = 0.28; p = 0.005), and SaO_2 _(r = -0.416; p = 0.000).

**Table 2:** Association between FPG and SaO_2 _among H1N1 and non-H1N1 patients with pneumonia

	**SaO_2_≧93%**		**SaO_2_<93%**	
	
	**FPG (mmol/L)**	**Hospitalization (days)**	**FPG (mmol/L)**	**Hospitalization (days)**
	
H1n1 group (n = 34)	6.64 ± 1.78	12.26 ± 7.63	9.82 ± 4.14**	19.22 ± 11.15*
Non-H1N1 group (n = 67)	6.02 ± 0.96	11.52 ± 10.88	7.81 ± 3.30	12.11 ± 7.56

*P < 0.05 vs.SaO_2_≧93% group; *significant negative correlation with SaO_2_

### 3. Influence of H1N1 infection on pancreatic islets function

Random determinations of FPG and FINS were made for suspected H1N1 patients on admission; information was collected for 21 confirmed-H1N1 patients and 34 non-H1N1 patients. The possible effects of H1N1 on pancreatic islets function was evaluated by calculating an insulin resistance index (homa-IR), an insulin function index(homa-β), and an insulin sensitivity index(IAI). The mean FPG level was significantly higher in the H1N1 group (8.29 ± 3.57 vs. 6.22 ± 1.62, H1N1 vs. non-H1N1; p < 0.05; Table 3). However, no statistically significant differences were found for homa-IR, homa-β, or IAI between the H1N1 and non-H1N1 groups after adjusting for age, gender, and BMI (Table 3).

**Table 3:** Comparison of blood glucose levels and pancreatic β-cells function between H1N1 and non-H1N1 patients

	Patient with non-H1N1 pneumonia (n = 31)	Patients with confirmed H1N1 pneumonia (n = 21)
FBG (mmol/L)	6.22 ± 1.62	8.29 ± 3.57*

FINS(μU/mL)	15.07 ± 6.72	14.96 ± 6.70

HbA1c (%)	5.83 ± 0.60	6.22 ± 1.78

Homa-IR	4.12 ± 1.98	5.27 ± 3.88

Homa-β	134.73 ± 76.76	103.13 ± 82.37

IAI	4.42 ± 0.49	4.61 ± 0.55

*P < 0.05 vs. non-H1N1 patients after controlling age, sex and BMI

### 4. Predictors for the severity and clinical course of H1N1

In the H1N1 group, the FPG levels of patients with hypoxemia (SaO_2 _<93%) were higher than those of patients without hypoxia (9.82 ± 4.14 vs. 6.64 ± 1.78; p < 0.05; Table 2). After adjusting for age, gender, and BMI, a significant negative correlation was found between FPG levels and SaO_2 _in the H1N1 group with SaO_2 _< 93%(r = -0.497; p = 0.041). The mean course of H1N1 patients with SaO_2 _≥ 93% was 12.26 ± 7.63 d, whereas for patients with SaO_2_<9 3% it was 19.22 ± 11.15 d; there was a significant difference between the 2 groups (p < 0.05). In addition to FPG, some biochemical indicators that might influence or predict the disease course were analyzed by linear regression analysis. These results showed that number of hospitalization days were correlated with LDH (β = 0.000297 ± 0.0000; p = 0.049), BUN (β = 0.0891 ± 0.036; p = 0.013), PaO_2 _(β = -0.0119 ± 0.004; p = 0.003), and SaO_2 _(β = -0.0287 ± 0.013; p = 0.036).

## Discussion

Since the global outbreak of H1N1 in 2009, a large number of patients have died of H1N1 complications. This virus can not only cause severe pneumonia and respiratory failure, but can also impair the myocardium, and hepatic and renal functions and can even lead to multiple organ failure (MOF) [11,12]. However, there have been no reports regarding whether H1N1 virus can affect pancreatic β-cells. In this paper, we retrospectively analyzed suspected and confirmed H1N1 cases and found that the FPG level of H1N1 patients on admission was an important and independent predictor for the severity of H1N1.

It has been reported that diabetes mellitus (DM) is an independent risk factor for H1N1 infection and that the mortality of H1N1 patients complicated with DM is much higher [4,5,13]. In our study, those H1N1 patients with a diabetic history or pregnancy were excluded so as to remove possible interference on FPG levels. For similar reasons, those cases that died were also excluded to remove confounding effects from the statistical analysis. Most of the patients in our study were cured and discharged after appropriate therapies, except for 2 cases that died.

Homa-IR, homa-β, and IAI are three important formulas for analyzing pancreatic β-cells function, and are widely used in the statistical analysis of clinical or basic research. They represent, respectively, insulin resistance, pancreatic β-cells function, and insulin sensitivity. Logistic regression analysis and t-tests both demonstrated notable relationships between FPG levels and H1N1 infection, and higher FPG levels in the H1N1 group than those in the non-H1N1 group. However, H1N1 pneumonia did not affect Homa-IR, homa-β, and IAI, which indicated that the H1N1 virus might not directly damage pancreatic β-cells function, and the elevated FPG levels in the H1N1 group might be an indirect result. In contrast, the trend for downregulated homa-β in the H1N1 group (Table2) indicates that H1N1 could potentially damage pancreatic β-cells.

Correlation analysis for H1N1 patients showed clear positive correlations between FPG and LDH, BUN and AST levels, whereas there was a clear negative correlation between FPG and SaO_2_. These results indicated changes in inflammation, renal function, hepatic function, and pulmonary function. We speculate that H1N1-mediated elevations of FPG might be the result of multiple factors' combinations caused by H1N1 virus infection, such as inflammatory stress, multiple organ impairment, and so on, while the changes of plasma glucose were not caused by H1N1 actions on insulin secretion by pancreatic β-cells or decreasing sensitivity to insulin.

To investigate the relationship between FPG and the severity of H1N1 pneumonia, we used SaO_2 _on admission and the hospitalization course as indicators. We set FPG of <5.6 mmol/L or ≥ 7 mmol/L as cut-off values for low and high FPG, respectively. According to the Chinese Ministry of Health criteria, hypoxia was defined as SaO_2 _< 93%, probably resulting from diffuse alveolar damage caused by H1N1 infection. In patients with obvious hypoxia (SaO_2 _< 93%), there was a significantly negative correlation between FPG and SaO_2_, indicating that FPG could be a predictor of severity in H1N1 patients with severe hypoxic conditions. Our results showed that FPG was not a predictor for the number of hospitalization days, although elevated FPG levels and a prolonged stay in the hospital were in parallel. Thus, we consider that the FPG level on admission is a preliminary and feasible indicator for predicting the severity of H1N1 pneumonia.

The H1N1 virus can cause damage to the body in various ways. In addition to extensive pulmonary inflammation and striking respiratory failure, it can also impair multiple organ systems [14,15]. Our results provide evidence that the myocardium, liver and kidneys are involved in H1N1 virus infection, as the myocardial enzymes, aminotransferases, and creatinine were elevated. Nevertheless, using the number of admission days as an indicator of the severity of H1N1 pneumonia, only LDH, BUN, PaO_2_, and SaO_2 _were suitable for assessing disease duration. These results indicated that FPG could not be used to predict the clinical course of H1N1 in the hospital, but could be used as a preliminary predictor for the severity of H1N1 infection.

However, our study has some limitations. 1. An elevated FPG level could be due to multiple factors in H1N1 patients, whereas we only analyzed the associations between FPG and biochemical indicators; 2.We did not determine correlations between pneumonia scoring systems and FPG levels; and 3. The limited data in our study requires additional verification.

## Conclusions

In this paper, we retrospectively analyzed cases of suspected and confirmed H1N1 infections in our hospital, and indicated the importance of FPG for evaluating the severity of H1N1 infection. Previous reports did not indicate whether the H1N1 virus had an influence on pancreatic β-cells function. Our results demonstrated that elevated FPG induced by H1N1 pneumonia could not be a result of direct damage to pancreatic β-cells, but could arise from various factors' combinations caused by H1N1 virus infection. The FPG level of H1N1 patients on admission is a simple and feasible indicator to predict the disease tendency; however, more information and clinical data are required to investigate whether the prognosis of H1N1 pneumonia will benefit from plasma glucose control and regulation.

## Abbreviations

BMI: body mass index; AST: aspartate aminotransferase; ALT: alanine aminotransferase; CK: creatine kinase; CK-MB: MB isoenzyme of creatine kinase; CREA: creatinine; BUN: urea nitrogen; LDH: lactate dehydrogenase; FPG: fasting plasma glucose; FINS: fasting plasma insulin; HbA1c: haemoglobin A1c; PO_2_: partial pressure of oxygen; PCO_2_: partial pressure of carbon dioxide; SO_2_: Oxygen Saturation.

## Competing interests

The authors declare that they have no competing interests.

## Authors' contributions

WW, HC, QL: Conceived and supervised the study. WW and HC contributed equally to this work, All the authors contributed to the writing and finalizing the manuscript.

## Pre-publication history

The pre-publication history for this paper can be accessed here:

http://www.biomedcentral.com/1471-2334/11/104/prepub

## References

[B1] Centers for Disease Control and Prevention (CDC) Bacterial coinfections in lung tissue specimens from fatal cases of 2009 pandemic influenza A (H1N1) - United States, May-August 2009 MMWR Morb Mortal Wkly Rep 2009 58 1071 4 19798021

[B2] Centers for Disease Control and Prevention (CDC) Outbreak of swine-origin influenza A (H1N1) virus infection-Mexico, March-April 2009 MMWR Morb Mortal WklyRep 2009 58 467 70 19444150

[B3] World Health Organization Influenza A (H1N1)-update 79 http://www.who.int/csr/don/2009_12_18a/en/index.html

[B4] Dee S Jayathissa S Clinical and epidemiological characteristics of the hospitalised patients due to pandemic H1N1 2009 viral infection: experience at Hutt Hospital, New Zealand N Z Med J 2010 123 45 53 20389317

[B5] Hanslik T Boelle PY Flahault A Preliminary estimation of risk factors for admission to intensive care units and for death in patients infected with A (H1N1)2009 influenza virus, France, 2009-2010 PLoS Curr Influenza 2010 RRN1150 10.1371/currents.RRN1150 PMC2836028 20228857

[B6] Barboza P Baudon C Chérié-Challine L Gastellu-Etchegorry M Gueguen J La Ruche G Grangeon JP Laumond-Barny S Noël M Pfannstiel A Chee-Ayee A Daudens E Frogier E Le B Mallet HP Pescheux JP Vergeaud H Lastère S Dutaut E Yvon JF Influenza A (H1N1)2009 in the French Pacific territories: assessment of the epidemic wave during the austral winter Clin Microbiol Infect 2010 16 304 8 20121824 10.1111/j.1469-0691.2010.03172.x

[B7] World Health Organization Human infection with pandemic (H1N1) 2009 virus: updated interim WHO guidance on global surveillance http://www.who.int/csr/resources/publications/swineflu/interim_guidance/en/

[B8] Ministry of Health of The People's Republic of China http://61.49.18.65/publicfiles/business/cmsresources/H1N1/cmsrsdocument/doc6806.doc

[B9] Monzillo LU Hamdy O Evaluation of insulin sensitivity in clinical practice and in research settings Nutr Rev 2003 61 397 412 10.1301/nr.2003.dec.397-412 14968910

[B10] Bonora E Targher G Alberiche M Bonadonna RC Saggiani F Zenere MB Homeostasis model assessment closely mirrors the glucose clamp technique in the assessment of insulin sensitivity: studies in subjects with various degrees of glucose tolerance and insulin sensitivity Diabetes Care 2000 23 57 63 10.2337/diacare.23.1.57 10857969

[B11] Louie JK Acosta M Winter K Jean C Gavali S Schechter R Vugia D Harriman K Matyas B Glaser CA Samuel MC Rosenberg J Talarico J Hatch D California Pandemic (H1N1) Working Group Factors associated with death or hospitalization due to pandemic 2009 influenza A(H1N1) infection in California JAMA 2009 302 1896 902 10.1001/jama.2009.1583 19887665

[B12] Fajardo-Dolci G Gutierrez-Vega R Arboleya-Casanova H Villalobos A Wilson KS García SG Sotelo J Córdova Villalobos JA Díaz-Olavarrieta C Clinical characteristics of fatalities due to influenza A (H1N1) virus in Mexico Thorax 2010 65 505 9 10.1136/thx.2009.126953 20522847

[B13] Allard R Leclerc P Tremblay C Tannenbaum TN Diabetes and the severity of pandemic influenza A (H1N1) infection Diabetes Care 2010 33 1491 3 10.2337/dc09-2215 20587722 PMC2890346

[B14] Kumar A Zarychanski R Pinto R Cook DJ Marshall J Lacroix J Stelfox T Bagshaw S Choong K Lamontagne F Turgeon AF Lapinsky S Ahern SP Smith O Siddiqui F Jouvet P Khwaja K McIntyre L Menon K Hutchison J Hornstein D Joffe A Lauzier F Singh J Karachi T Wiebe K Olafson K Ramsey C Sharma S Dodek P Meade M Hall R Fowler RA Canadian Critical Care Trials Group H1N1 Collaborative Critically ill patients with 2009 influenza A(H1N1) infection in Canada JAMA 2009 302 1872 9 10.1001/jama.2009.1496 19822627

[B15] Domínguez-Cherit G Lapinsky SE Macias AE Pinto R Espinosa-Perez L de la Torre A Poblano-Morales M Baltazar-Torres JA Bautista E Martinez A Martinez MA Rivero E Valdez R Ruiz-Palacios G Hernández M Stewart TE Fowler RA Critically Ill patients with 2009 influenza A(H1N1) in Mexico JAMA 2009 302 1880 7 10.1001/jama.2009.1536 19822626

